# Supplementary material for: Early human impact on lake cyanobacteria revealed by a Holocene record of sedimentary ancient DNA
Source: Commun Biol. 2023 Jan 18;6:72. doi: 10.1038/s42003-023-04430-z (PMC9849356; doi:10.1038/s42003-023-04430-z)
Supplement: Supplementary file 2 — Supplementary Infomation [file 42003_2023_4430_MOESM2_ESM.pdf]

Supplementary Information to manuscript “Early human impact on lake cyanobacteria revealed by a Holocene record of sedimentary ancient DNA”

Authors: Ebuka Canisius Nwosu*, Achim Brauer, Marie-Eve Monchamp, Sylvia Pinkerneil, Alexander Bartholomäus, Martin Theuerkauf, Jens-Peter Schmidt, Kathleen R. Stoof-Leichsenring, Theresa Wietelmann, Jerome Kaiser, Dirk Wagner, and Susanne Liebner

*Corresponding Author's email: enwosu@gfz-potsdam.de

Supplementary Note 1. DNA extraction for shotgun sequencing.

The DNA for shotgun (metagenomic) sequencing was extracted using DNeasy PowerMax Soil DNA and the DNeasy Power Soil DNA 100 DNA isolation kits (Qiagen) from 14 sediment samples distributed from top to bottom of the long core (Supplementary Table 2). For this, total DNA was extracted from the 12 samples weighing between 0.7–2.6 g wet sediment. Two DNA extraction kits were used in the paleogenetic laboratories at Alfred Wegener Institute (AWI) Potsdam inside a UV-cleaner box. To avoid contamination, the solutions C2, C3, and C4, which were only used on the second day of extraction, were prepared before working with the sediment samples. The extraction protocols were modified as previously described[1]. Briefly, prior to adding the sediment sample, solution C1, 400 µL of proteinase K (2 mg ml^{-1}) and 100 µL of Dithiothreitol (DTT) (5M) were added and vortexed for 10 min. Afterward, the tubes containing the samples and the control were placed into a rotating oven set to 56 °C overnight. The extracted DNA was eluted with 2 mL of provided elution buffer. Two extractions were performed for each depth and the extracts of the same sediment sample were pooled for the DNA concentration and purification steps. The elution of the extracts was done with 100 µL of the provided elution buffer. The DNA concentration was determined using a Qubit dsDNA broad-range assay kit and the Qubit R 2.0 Fluorometer. Every sample was measured twice and the average was calculated. Such concentration analyses were conducted before concentrating and purifying the extracted DNA as well as

afterward. To concentrate and purify the sedaDNA extracts, the GeneJET PCR purification kit (ThermoFisher) was used following the manufacturer's instructions. The samples extracted from the same depth were pooled and concentrated together. The elution volume varied from 50 µL to 100 µL depending on the previously measured DNA concentration.

Cyanobacteria lipid biomarker analysis

Lipid biomarker analysis of 7Me-$C_{17:0}$ monomethyl branched alkanes (7-methylheptadecane) are produced mainly by heterocystous, ramified, and some filamentous cyanobacteria[2–4] and might be representative of cyanobacterial biomass as recently shown for the Baltic Sea[5]. Sediment samples (n = 42) dating back to ca. 6,180 cal. a BP from a parallel core correlated to the reference core based on varve counting, tephrochronology, and radiocarbon dating[6] were analyzed following[5,7]. Sediments (0.4 g dry weight) were extracted with a mixture of DCM/MeOH (9:1) and accelerated solvent extraction (Dionex ASE 350). Squalane was added as an internal standard. The apolar lipid fractions containing the hydrocarbons were obtained by column chromatography (SiO_2) using hexane as eluent. The fractions were analyzed by gas chromatography–mass spectrometry (GC–MS) using an Agilent Technologies 7890B GC system coupled to a 5977B Mass Selective Detector equipped with an HP-5 ms capillary column (30 m * 0.25 mm * 0.25 lm). The oven temperature was programmed from 40 to 320 °C at 8 °C min^{-1} followed by a 15 min isotherm. GC–MS data were collected in total ion current (TIC) (m/z 50–600). The fractions were also analyzed using a multichannel TraceUltra gas chromatograph (ThermoScientific) equipped with a DB-5 ms capillary column (30 m * 0.25 mm * 0.25 lm) and a flame ionization detector (FID). The temperature program was identical to that used for analysis by GC–MS and peak identification was made by comparison of retention times using the two methods. For quantification, the GC-FID response of each lipid was normalized to that of the internal standard and the amount of

sediment extracted. The detection limit was estimated to ca. 4–5 ng g^{-1}. The data were further normalized to total organic carbon (TOC).

Supplementary Note 2. Analysis of cyanobacterial DNA preservation

The analysis of cyanobacterial DNA preservation resulted in successful short fragment amplicons for the sediment depths 265 (20 clones; ca. 813 cal. a BP), 490 (19 clones; ca. 2,385 cal. a BP), and 1,105 cm (19 clones; ca. 10,780 cal. a BP; Supplementary Table 3). The picocyanobacteria (*Synechococcus* and *Cyanobium*) made up the majority of the sequenced clones with mean sequence similarity ranging between 91–100%. Long fragment amplicons were only obtained from sediments dated to ca. 813 cal. a BP (20 clones) and ca. 2,385 cal. a BP (19 clones). The composition of cyanobacterial assemblages was similar (*Synechococcus*) regardless of amplicon length in the sample dated to ca. 813 cal. a BP. The long amplicon sequences from ca. 2,385 cal. a BP were dominated by *Leptolyngbya* and *Pseudanabaena*. These results confirmed that long and short cyanobacterial DNA fragments were well preserved along the entire sediment core (11 m).

Cyanobacterial lipid biomarker reconstructed from sediments

The TOC normalized content of 7Me-$C_{17:0}$ ranged between ~ 0.05 and 49 µg g^{-1} TOC. A maximum content occurred in CE 2000 (33.1 µg g^{-1} TOC) that correlated with the highest relative abundance of ASVs assigned to *Aphanizomenon* (Supplementary Fig. 6). The lowest value (0.05 µg g^{-1} TOC) was measured in a sample corresponding to the year (CE 1830). The mean content of 7-Methylheptadecane in samples (n = 6) corresponding to early to mid-Holocene was 1.7 µg g^{-1} TOC. In samples (n = 6) from Bronze Age to the Little Ice Age the mean value increased slightly to 1.9 µg g^{-1} TOC. Only in modern strata (n = 15) was 7-Methylheptadecane values substantially higher 8.4 µg g^{-1} TOC.

Supplementary Table 1. Sediment sample characteristics: SedaDNA concentration, chronology in calibrated age before present and common/before common era (BC/BCE), sedaDNA normalized to sediment quantity used for extraction and TOC.

Sample ID	Sediment	DNA conc. (ng µl)	Age cal. a BP (= before 1950)	Age CE/BCE	TOC %	Sed. Qty (g)	µg DNA/g Sed.	µg DNA/TOC
	composite depth (cm)							
H1_1-2	3	37.1	-50	2000	11	1.1	3.4	0.33
H1_2-3	5	34.6	-45	1995	9	1.0	3.4	0.38
H1_3-4	7	32.5	-40	1990	9	0.7	4.4	0.34
H1_4-5	8	28.5	-36	1986	10	1.0	2.8	0.28
H1_5-6	10	33.2	-33	1983	8	1.1	3.0	0.41
H1_6-7	11	20.7	-30	1980	10	1.0	2.0	0.22
H1_7-8	13	22.8	-27	1977	8	1.0	2.2	0.28
H1_8-9	14	18.8	-24	1974	11	1.0	1.9	0.18
H1_9-10	16	21.8	-20	1970	9	1.2	1.9	0.23
H1_10-11	17	14.8	-15	1965	9	1.0	1.4	0.17
H1_11-12	19	20.6	-13	1963	8	1.1	1.9	0.27
H1_15-16	24	15.6	-2	1952	8	1.0	1.6	0.19
H1_20-21	31	16.1	10	1940	8	1.0	1.6	0.21
H1_25-26	37	12.5	20	1924	8	1.1	1.2	0.15
H1_30-31	38	8.54	30	1920	9	1.0	0.8	0.10
H1_35-36	44	7.4	60	1890	11	1.0	0.7	0.07
H1_40-41	48	3.7	80	1870	9	1.0	0.4	0.04
H1_45-46	52	8.48	100	1850	9	1.0	0.8	0.10
H1_50-51	55	7.13	110	1840	8	1.1	0.7	0.08
H1_55-56	57	5.97	120	1830	8	1.0	0.6	0.07
H1_60-61	61	8.98	140	1810	8	1.0	0.9	0.11
H1_65-66	65	4.9	160	1790	7	1.0	0.5	0.07
K1_28-29	184	9.83	530	1420	6	0.8	1.3	0.15
K1_38-39	194	8.53	560	1380	7	0.7	1.1	0.12
K1_48-49	204	8.43	590	1350	7	0.7	1.1	0.12
K1_58-59	214	11.2	630	1320	9	0.8	1.5	0.13
K1_68-69	224	14.8	660	1300	4	1.0	1.5	0.36
K1_78-79	234	17.2	690	1260	7	1.0	1.7	0.26
K1_88-89	244	14.4	720	1230	5	1.0	1.4	0.29
H2_10-11	265	29	810	1140	10	0.8	3.9	0.29
H2_15-16	270	15.7	850	1100	14	0.8	2.1	0.11
H2_20-21	275	12.1	880	1070	14	0.7	1.6	0.08
H2_40-41	295	10.9	1010	940	16	0.8	1.5	0.07
H2_60-61	315	10.9	1150	800	19	0.7	1.5	0.06
H2_80-81	335	15	1280	670	15	0.7	2.0	0.10
H2_95-96	350	16	1380	570	18	1.0	1.6	0.09
H2_105-106	360	14.5	1450	500	16	1.0	1.4	0.09
H2_120-121	375	8.96	1550	400	17	1.0	0.9	0.05

Sample ID	Sediment composite depth (cm)	DNA conc. (ng/µl)	Age cal. a BP (= before 1950)	Age CE/BCE	TOC	Sed. Qty (g)	µg DNA/g Sed.	µg DNA/TOC
H2_125-126	380	15.8	1580	370	16	1.0	1.6	0.10
H2_130-131	385	15.9	1620	340	17	1.0	1.6	0.09
H2_135-136	390	18.3	1650	300	16	1.0	1.8	0.12
H2_140-141	395	12.5	1690	270	15	1.0	1.3	0.08
H2_145-146	400	9.49	1710	230	16	1.0	0.9	0.06
H2_150-151	405	8.55	1750	200	14	1.0	0.9	0.06
H2_175-176	430	16.6	1900	50	19	1.0	1.7	0.09
H2_185-186	441	15	1960	-10	13	1.0	1.5	0.11
K2_108-109	464	13.9	2100	-150	17	1.0	1.4	0.08
H3_10-11	490	7.45	2380	-440	17	1.0	0.8	0.04
H3_35-36	516	4.61	2610	-660	15	1.0	0.5	0.03
H3_40-41	521	6.08	2660	-710	16	1.0	0.6	0.04
H3_55-56	534	4.74	2800	-850	11	1.0	0.5	0.04
H3_75-76	554	8.12	2980	-1050	15	1.0	0.8	0.05
H3_95-96	574	4.38	3190	-1260	15	1.0	0.4	0.03
H3_110-111	589	2.07	3360	-1420	6	1.1	0.2	0.04
H3_125-126	604	2.85	3520	-1580	6	0.9	0.3	0.05
H3_140-141	619	1.65	3690	-1750	4	1.0	0.2	0.04
H3_165-166	644	5.55	3960	-2020	17	1.0	0.5	0.03
H3_175-176	654	9.44	4070	-2120	15	0.9	1.0	0.06
H4_50-51	747	6.54	5070	-3120	18	1.1	0.6	0.04
H4_75-76	769	3.41	5260	-3310	14	1.0	0.3	0.03
H4_100-101	794	4.12	5450	-3500	14	1.0	0.4	0.03
H4_125-126	820	3.84	5690	-3740	20	1.0	0.4	0.02
H4_150-151	848	2.06	6180	-4080	16	1.0	0.2	0.01
H4_175-176	872	2.72	6580	-4400	22	1.0	0.3	0.01
H4_190-191	887	1.16	6840	-4590	19	1.0	0.1	0.01
H5_20-21	927	0.75	7530	-5450	20	0.9	0.1	0.00
H5_45-46	954	0.8	7990	-6060	19	1.0	0.1	0.00
H5_70-71	981	1	8470	-6550	15	1.0	0.1	0.01
H5_95-96	1012	1.19	9020	-7110	11	1.0	0.1	0.01
H5_120-121	1033	0.902	9380	-7520	16	1.0	0.1	0.01
H5_145-146	1058	0.657	9830	-7980	11	1.0	0.1	0.01
H5_170-171	1089	0.874	10,430	-8530	12	1.0	0.1	0.01
H5_185-186	1105	1.16	10,830	-8830	12	1.0	0.1	0.01
H6_15-16	1126	0.903	11,340	-9270	1	1.0	0.1	0.06

The sediment age chronology up to ca. 6,000 cal. a BP for Lake Tiefer See is published at https://doi.org/10.1177/0959683616660173.

Supplementary Table 2: Total cyanobacteria abundance determined via qPCR, and cyanobacteria lipid biomarker 7-methylheptadecane normalized over total organic carbon (TOC).

Sample ID	Total Cyanobacteria abundance 16S rRNA-ITS	7-Methylheptadecane ($\mu g\ g\ TOC^{-1}$)	Sample ID	Total Cyanobacteria abundance 16S rRNA-ITS	7-Methylheptadecane ($\mu g\ g\ TOC^{-1}$)
H1_1-2	8.26E+04	33.05	H2_125-126	1.44E+03	
H1_2-3	2.09E+05	0.31	H2_130-131	5.80E+03	
H1_3-4	1.45E+05	48.97	H2_135-136	2.37E+03	2.21
H1_4-5	8.52E+04	0.42	H2_140-141	1.45E+05	
H1_5-6	9.26E+04	0.46	H2_145-146	5.82E+04	
H1_6-7	7.49E+04	0.49	H2_150-151	2.18E+04	1.56
H1_7-8	7.71E+04	0.44	H2_175-176	1.66E+04	0.76
H1_8-9	7.43E+04	0.42	H2_185-186	1.59E+04	1.89
H1_9-10	6.66E+04	0.46	K2_108-109	2.89E+04	3,98
H1_10-11	7.78E+04	0.62	H3_10-11	1.33E+04	
H1_11-12	6.19E+04	0.48	H3_35-36	6.50E+03	
H1_15-16	9.42E+04	24.11	H3_40-41	7.85E+03	6.80
H1_20-21	4.82E+04	4.72	H3_55-56	3.70E+03	
H1_25-26	6.47E+04	10.06	H3_75-76	2.59E+03	0.47
H1_30-31	2.40E+04		H3_95-96	1.57E+03	
H1_35-36	1.44E+04	3.08	H3_110-111	1.40E+03	
H1_40-41	1.85E+04		H3_125-126	3.18E+03	
H1_45-46	2.32E+04	1.88	H3_140-141	2.21E+03	1.88
H1_50-51	2.14E+04	0.20	H3_165-166	5.42E+02	2.41
H1_55-56	1.99E+04	0.05	H3_175-176	1.23E+03	1.24
H1_60-61	8.96E+03		H4_50-51	6.07E+02	2.96
H1_65-66	1.13E+04	0.19	H4_75-76	3.70E+02	2.40
K1_28-29	2.63E+04		H4_100-101	3.55E+02	0.79
K1_38-39	1.32E+04		H4_125-126	7.16E+02	1.13
K1_48-49	2.76E+04	0.71	H4_150-151		1.42
K1_58-59	3.13E+04	0.41	H4_175-176	8.73E+02	
K1_68-69	2.77E+04		H4_190-191		
K1_78-79	1.62E+04		H5_20-21	7.07E+02	
K1_88-89	2.38E+04	0.48	H5_45-46	1.17E+02	
H2_10-11	8.37E+04	2.60	H5_70-71	1.37E+02	
H2_15-16	3.13E+03	4.00	H5_95-96		
H2_20-21	6.14E+03	1.39	H5_120-121	5.24E+02	
H2_40-41	9.14E+03		H5_145-146		
H2_60-61	1.20E+04		H5_170-171		
H2_80-81	9.50E+03		H5_185-186	8.07E+02	
H2_95-96	1.69E+04	3.31	H6_15-16	2.22E+02	
H2_105-106	9.87E+03	3.09			
H2_120-121	8.25E+03				

Supplementary Table 2 cont.: Cyanobacteria amplicon sequence data, cyanobacteria ASV (taxonomic) richness and shotgun (metagenome) sequencing data.

Sample ID	Amplicon DADA2 Pipeline sequence data			Cyanobacteria ASV richness	Shotgun sequencing	
	Input	Processed	final reads		DNA conc. (µg)	Read counts
H1_1-2	190740	72119	50860	169		
H1_2-3	193995	76383	54106	149		
H1_3-4	177211	69737	49975	142		
H1_4-5	203517	75436	55116	129	3.5	29,652,699
H1_5-6	149892	52269	36940	122		
H1_6-7	210433	76204	55287	124		
H1_7-8	143140	50326	37929	105		
H1_8-9	161329	58610	43274	102		
H1_9-10	197884	60059	53853	113	6.7	32,992,166
H1_10-11	213174	81189	63998	99		
H1_11-12	113461	41063	30857	66		
H1_15-16	110832	41386	31665	76		
H1_20-21	167987	62149	50238	84	6.7	46,135,529
H1_25-26	178452	66129	49618	77		
H1_30-31	127694	44684	35867	53		
H1_35-36	146271	51423	41399	46		
H1_40-41	123983	41705	34808	42		
H1_45-46	145207	52076	41894	51		
H1_50-51	93337	34445	29000	45		
H1_55-56	96120	35334	29184	49		
H1_60-61	124951	48854	42838	54		
H1_65-66	126327	50682	45091	47		
K1_28-29	43546	17362	14639	38		43,159,479
K1_38-39	41772	17272	14542	31		
K1_48-49	57033	23457	20373	38		
K1_58-59	52654	22097	19064	37		
K1_68-69	269639	110322	95607	51		
K1_78-79	149955	63433	54722	41		
K1_88-89	181172	75516	64565	49		
H2_10-11	64246	26137	22181	49	17.1	35,268,604
H2_15-16	69454	29407	25348	33		
H2_20-21	119596	51931	45533	47		30,419,097
H2_40-41	131166	56482	49522	63		
H2_60-61	98812	41745	34714	76	2.8	36,115,008
H2_80-81	104427	42508	36288	75	4.5	33,803926
H2_95-96	118718	53821	46285	61		
H2_105-106	140183	67023	57810	46		
H2_120-121	133572	63326	58743	36		
H2_125-126	127681	58406	50711	65		

Sample ID	Amplicon DADA2 Pipeline sequence data			Cyanobacteria ASV richness	Shotgun sequencing	
	Input	Processed	final reads		DNA conc. (µg)	Raw read counts
H2_130-131	148675	65925	59513	63		
H2_135-136	173727	77744	69317	90		
H2_140-141	92200	40701	35855	66		
H2_145-146	40873	18993	17056	33		
H2_150-151	61687	28363	24760	51		
H2_175-176	83830	36093	31879	59	3.4	48,549,277
H2_185-186	50503	21905	18836	51		
K2_108-109	226915	99393	86999	64		
H3_10-11	59084	28927	26347	31		
H3_35-36	45463	21783	20040	16		
H3_40-41	71077	34379	30787	23		29,498,111
H3_55-56	127106	61546	56644	17		
H3_75-76	150072	71055	63173	22		
H3_95-96	166682	81369	75546	25		
H3_110-111	171428	82996	78000	22		
H3_125-126	93537	43937	39660	14	1.2	22,561,531
H3_140-141	88424	42003	38569	14		
H3_165-166	143820	66888	61894	21		
H3_175-176	184536	79270	73718	45		
H4_50-51	168242	71088	65951	40	2.6	7,737,446
H4_75-76	157310	71616	65368	25		
H4_100-101	136266	59377	53738	19		
H4_125-126	179962	78643	72998	19		
H4_150-151	119253	51744	47949	16		
H4_175-176	142164	60129	56130	22		
H4_190-191	144009	67404	64139	14		
H5_20-21	147074	67633	65105	22	8.6	2,087,930
H5_45-46	90106	41205	39805	7		
H5_70-71	84010	38887	36357	10		
H5_95-96	125798	57595	54945	18		
H5_120-121	122113	58852	56323	24		
H5_145-146	99336	47416	45707	18		
H5_170-171	38788	17195	16416	14		
H5_185-186	35024	14926	14542	13		
H6_15-16	36260	16571	15829	14	1.4	114,185

Supplementary Table 3

Cyanobacterial composition of the long (~ 1500 bp) amplicons with short to medium amplicons (qPCR amplification products ~ 350 bp) from three sediment depths: 265 cm (H2_10-11), 490 cm (H3_10-11), and 1,105 cm (H5_185-186).

**Long fragments**		Top BLASTn Hit	Perc. Iden.			Top BLASTn Hit	Perc. Iden.
**265 cm**	H2_L_B1	*Synechococcus*	99%	**490 cm**	H3_L_A7	No result	
	H2_L_C1	*Synechococcus*	96%		H3_L_A8	*Leptolyngbya*	75%
	H2_L_D1	*Synechococcus*	99%		H3_L_C8	*Leptolyngbya*	74%
	H2_L_G2	*Synechococcus*	95%		H3_L_G7	*Pseudanabaena*	80%
	H2_L_H2	*Synechococcus*	97%		H3_L_H7	*Leptolyngbya*	76%
	H2_L_B3	*Synechococcus*	98%		H3_L_H8	*Pseudanabaena*	78%
	H2_L_F3	*Synechococcus*	98%		H3_L_C9	*Pseudanabaena*	80%
	H2_L_C3	*Synechococcus*	96%		H3_L_D9	*Pseudanabaena*	77%
	H2_L_G3	*Synechococcus*	97%		H3_L_B10	*Spirulina*	74%
	H2_L_D3	*Synechococcus*	98%		H3_L_D10	*Leptolyngbya*	80%
	H2_L_A4	*Synechococcus*	96%		H3_L_E10	*Pseudanabaena*	79%
	H2_L_A5	*Synechococcus*	98%		H3_L_E11	*Pseudanabaena*	79%
	H2_L_B4	*Synechococcus*	98%		H3_L_G11	No result	
	H2_L_B5	*Synechococcus*	99%		H3_L_H11	*Pseudanabaena*	78%
	H2_L_C4	*Synechococcus*	98%		H3_L_A12	No result	
	H2_L_D4	*Synechococcus*	98%		H3_L_E12	No result	
	H2_L_E5	*Synechococcus*	99%		H3_L_F12	*Phormidium*	75%
	H2_L_G6	*Synechococcus*	99%		H3_L_G12	*Pseudanabaena*	77%
	H2_L_H6	*Synechococcus*	99%		H3_L_H12	No result	
		**Average**	**98%**			**Average**	**77%**

**Short Fragments**		**Top BLASTn Hit**	**Perc.Iden.**			Top BLASTn Hit	Perc.Iden.
**265 cm**	H2_S_A1	*Synechococcus*	95%	**490 cm**	H3_S_A1	*Cyanobium*	89%
	H2_S_A2	*Synechococcus*	94%		H3_S_A2	*Synechococcus*	96%
	H2_S_B1	*Synechococcus*	94%		H3_S_B1	*Synechococcus*	90%
	H2_S_B2	*Synechococcus*	96%		H3_S_B2	*Synechococcus*	88%
	H2_S_C1	*Synechococcus*	100%		H3_S_C1	*Cyanobium*	89%
	H2_S_C2	*Synechococcus*	94%		H3_S_C2	*Cyanobium*	89%
	H2_S_D1	*Synechococcus*	96%		H3_S_D2	*Synechococcus*	96%
	H2_S_D2	*Synechococcus*	96%		H3_S_E2	*Cyanobium*	90%
	H2_S_F1	*Synechococcus*	94%		H3_S_F1	*Cyanobium*	89%
	H2_S_F2	*Synechococcus*	96%		H3_S_F2	*Cyanobium*	89%
	H2_S_A3	*Synechococcus*	96%		H3_S_G1	*Synechococcus*	88%
	H2_S_E3	*Synechococcus*	96%		H3_S_G2	*Nodosilinea*	90%
	H2_S_C3	*Synechococcus*	95%		H3_S_H1	*Synechococcus*	89%
	H2_S_G3	*Synechococcus*	96%		H3_S_H2	*Synechococcus*	87%
	H2_S_D3	*Synechococcus*	94%		H3_S_E3	*Synechococcus*	94%
	H2_S_H3	*Synechococcus*	97%		H3_S_B3	*Synechococcus*	88%
	H2_S_A4	*Synechococcus*	95%		H3_S_F3	*Cyanobium*	89%
	H2_S_A5	*Synechococcus*	94%		H3_S_D3	*Cyanobium*	91%
	H2_S_B4	*Synechococcus*	94%		H3_S_H3	*Cyanobium*	100%
	H2_S_C4	*Cyanobium*	96%			**Average**	**91%**
		**Average**	**95%**				
		**Top BLASTn Hit**	**Perc.Iden.**				
**1105 cm**	H5_S_B8	*Synechococcus*	95%				
	H5_S_E7	*Synechococcus*	89%				
	H5_S_F7	*Synechococcus*	95%				
	H5_S_F8	*Cyanobium*	97%				
	H5_S_G7	*Cyanobium*	96%				
	H5_S_G8	*Synechococcus*	95%				
	H5_S_H7	*Synechococcus*	96%				
	H5_S_E9	*Synechococcus*	89%				
	H5_S_F9	*Synechococcus*	96%				
	H5_S_C9	*Synechococcus*	95%				
	H5_S_D9	*Synechococcus*	95%				
	H5_S_A10	*Cyanobium*	96%				
	H5_S_E11	*Synechococcus*	95%				
	H5_S_F11	*Synechococcus*	96%				
	H5_S_G10	*Synechococcus*	95%				
	H5_S_G11	*Synechococcus*	95%				
	H5_S_A12	*Synechococcus*	96%				
	H5_S_C12	*Cyanobium*	96%				
	H5_S_D12	*Synechococcus*	96%				
		**Average**	95%				

Supplementary Table 4

Test results for Collinearity in the explanatory variables via variance inflation factor (VIF).

TOC	$\delta^{13}C_{OM}$	RecVegOp	Varve Quality	Years/Sample
1.753536	2.262912	2.053130	2.161087	1.872460

Supplementary Table 5

Rank-based Spearman correlation of cyanobacteria amplicon sequence variants (ASVs) with lake environmental parameters in Lake Tiefer See. The permutation *p*-values are (values in bold are significant at *p*-values < 0.05) above the diagonal and the corresponding F-values below. Values in blue show a positive correlation while red values show a negative correlation. TOC = Total organic carbon. RecVegOp = reconstructed vegetation openness. DNA concentrations are expressed as µl per gram sediment (DNA g^{-1} sed.) and µl over percentage of TOC (DNA TOC^{-1}).

**Spearman Correlation**	ASV Richness	16S rRNA-ITS	TOC	RecVegOp	Varve Quality	Years/Sample	DNA g^{-1} Sed.	DNA TOC^{-1}
ASV Richness		**0.003**	0.1	0.1	**0.003**	**0.003**	**0.003**	**0.003**
16S rRNA-ITS	0.73764		**0.003**	**0.003**	0.1	**0.003**	**0.003**	**0.003**
TOC	-0.17419	-0.46818		**0.003**	**0.02**	**0.003**	1	**0.003**
RecVegOp	0.2604	0.49799	-0.59826		**0.02**	**0.004**	**0.4**	**0.007**
Varve Quality	0.49581	0.2056	0.26969	-0.35345		0.16061	**0.003**	0.4
Years/Sample	-0.68766	-0.77357	0.48706	-0.43326	-0.16479		**0.003**	**0.003**
DNA g^{-1} Sed.	0.86	0.65	-0.16	0.39	0.49	-0.66		**0.003**
DNA TOC^{-1}	0.82	0.76	-0.53	0.58	0.29	-0.7	0.89	

Supplementary Table 6

Overall test statistics of the one-way PerMANOVA on cyanobacteria assemblage from amplicon sequencing grouped into three time periods: (I) ca. 11,340–4,070 cal. a BP, (II) ca. 3,960–100 cal. a BP, and (III) CE 1870–2000. Pairwise analysis shows Bonferroni corrected significant *p*-values (in bold) above the diagonal and corresponding *F*-values below.

Permutation N:	9,999				
Total sum of squares:	27.39	**Pairwise**	**III**	**II**	**I**
Within-group sum of squares:	22.74	**III**		**0.0003**	**0.0003**
F:	6.952	**II**	9.412		**0.0003**
p (same):	0.0001	**I**	7.246	4.52	

Supplementary Figure 1

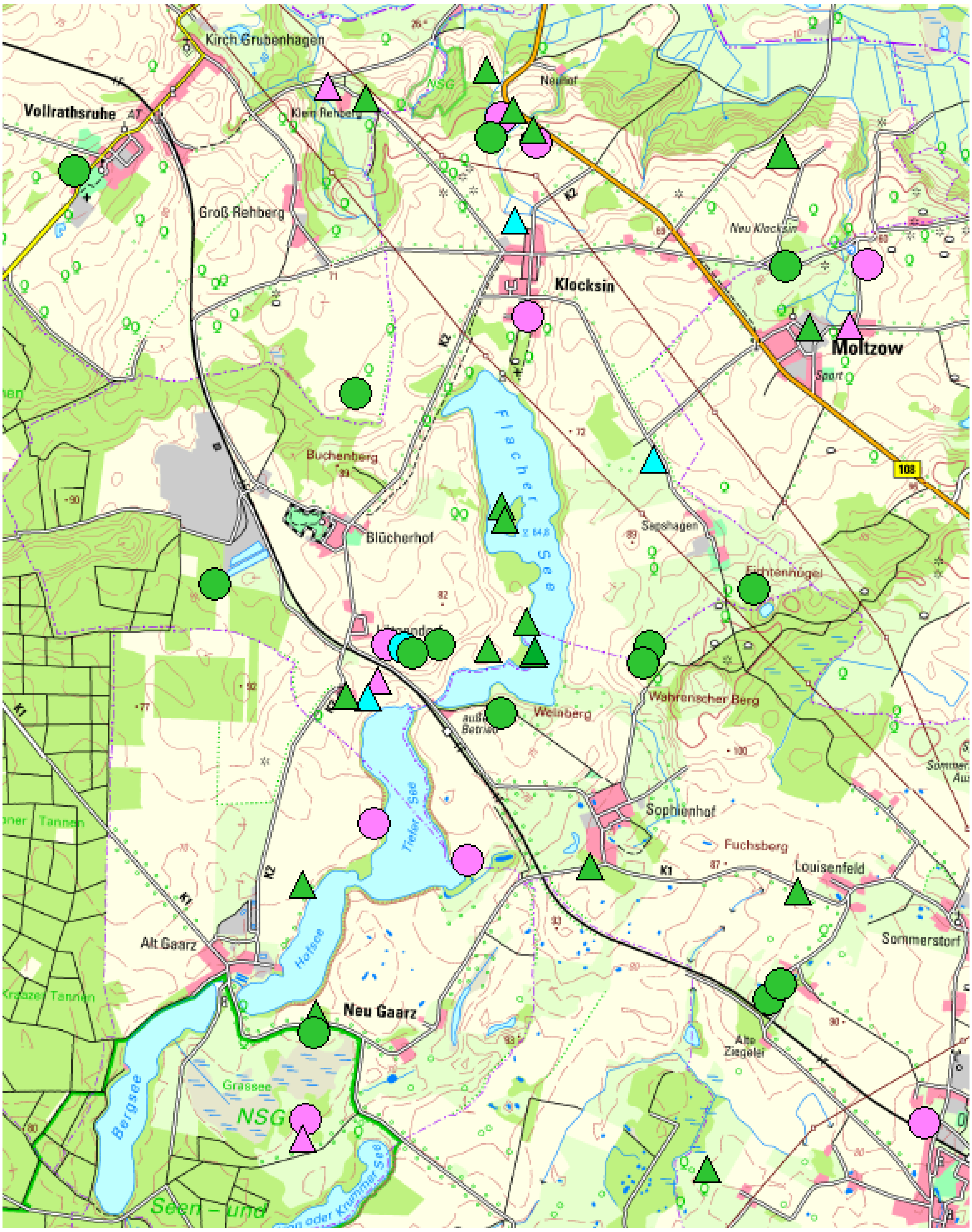

Mapping of Bronze Age and Roman Iron Age sites of archaeological finds in the vicinity of Lake Tiefer See. Green circles denote burial sites and settlements from the Bronze Age, blue and pink circles denote settlements from the pre-Roman Iron Age (ca. BCE 550–0) and the Roman Iron Age Period (ca. CE 0–400), respectively. Triangles represent scattered finds, individual finds, deposits (that is, victims) and cup-marked stones.

Supplementary Figure 2

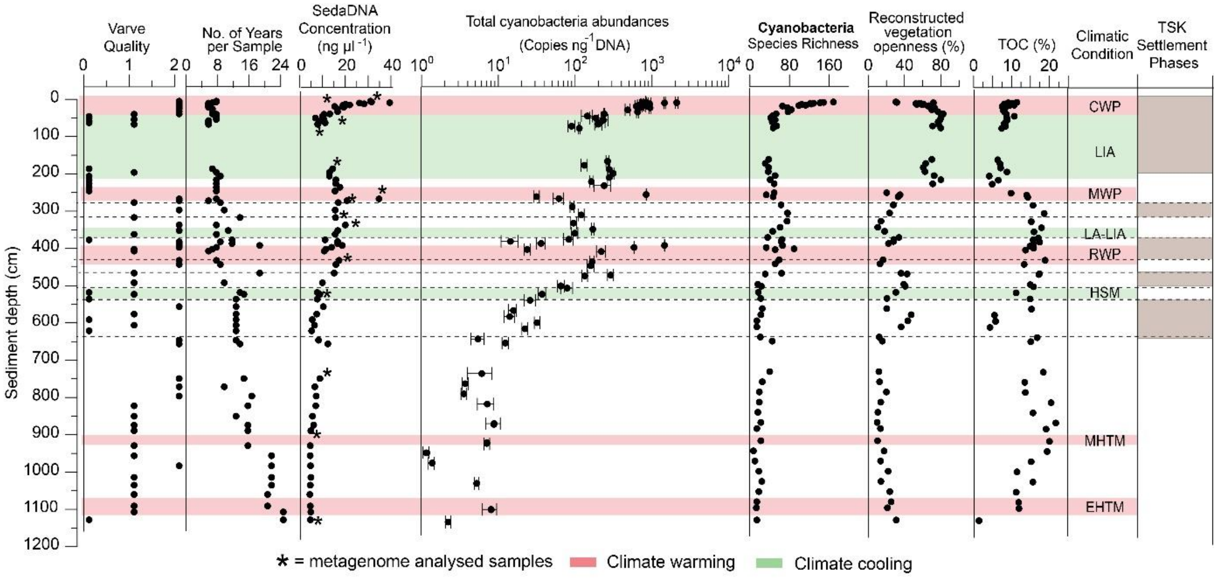

Sediment composite profile (cm) with sedimentological parameters, sedaDNA concentration, cyanobacteria data, and selected lake paleoenvironmental records. Sedimentological analysis: varve quality (VQ; 0 = non-varved, 1 = poorly-, and 2 = well-varved sediment samples) and the number of years in each analyzed sediment sample. Microbiological analysis: sedaDNA concentration); total cyanobacteria abundance determined via quantitative PCR (error bars give the standard deviations for three independent amplifications), cyanobacteria ASV richness from amplicon sequencing. Geochemical analyses: TOC contents. Pollen-based reconstructed vegetation openness data. The climatic conditions (warmer periods; red. and cooler periods; light green) are based on Wanner et al. 2015, and Büntgen et al. 2016. The Tiefer See near Klocksin (TSK) settlement phases are derived from vegetation openness data. CWP = current warming period. LIA = Little Ice Age. MWP = Medieval Warm Period. LA-LIA = Lat antique Little Ice Age (Dark Ages). RWP = Roman Warm Period. HSM = Homeric Solar Minimum. MHTM = Mid-Holocene Temperature Maximum. and EHTM = Early Holocene Temperature Maximum

Supplementary Figure 3

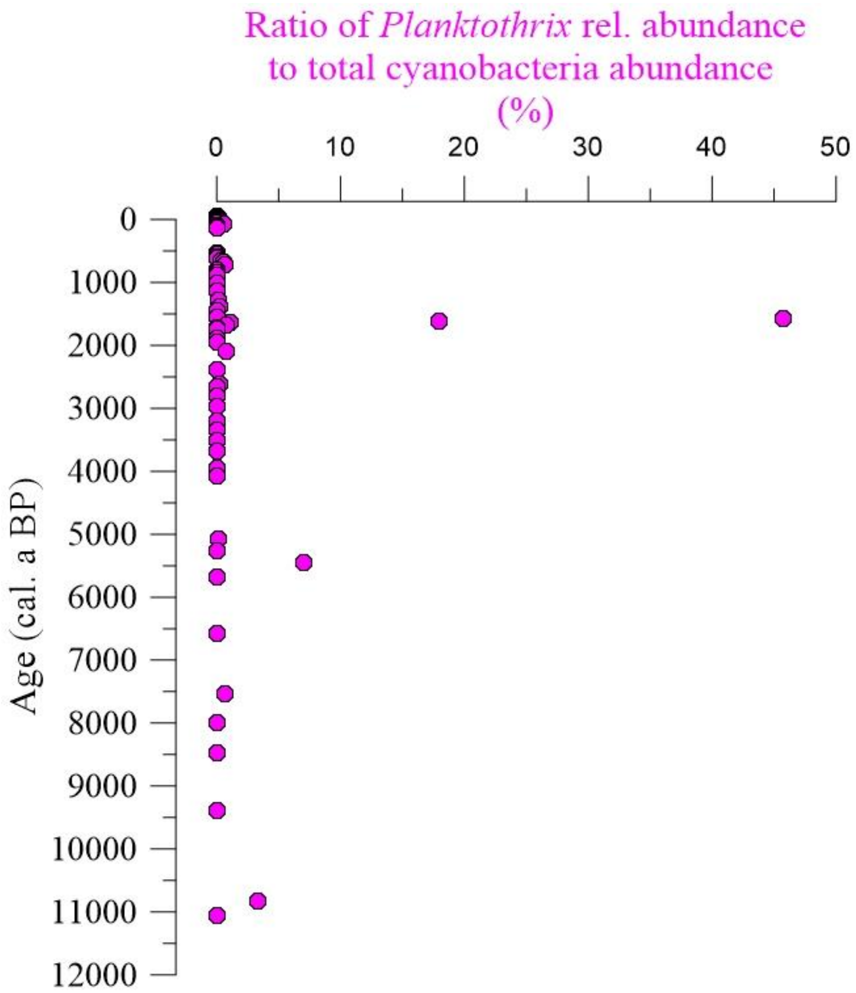

Test for bias related to differential DNA preservation within sediment layers was done by calculating the ratio of relative abundance of *Planktothrix* ASVs obtained from amplicon sequencing to the total cyanobacteria abundance estimated by qPCR.

Supplementary Figure 4

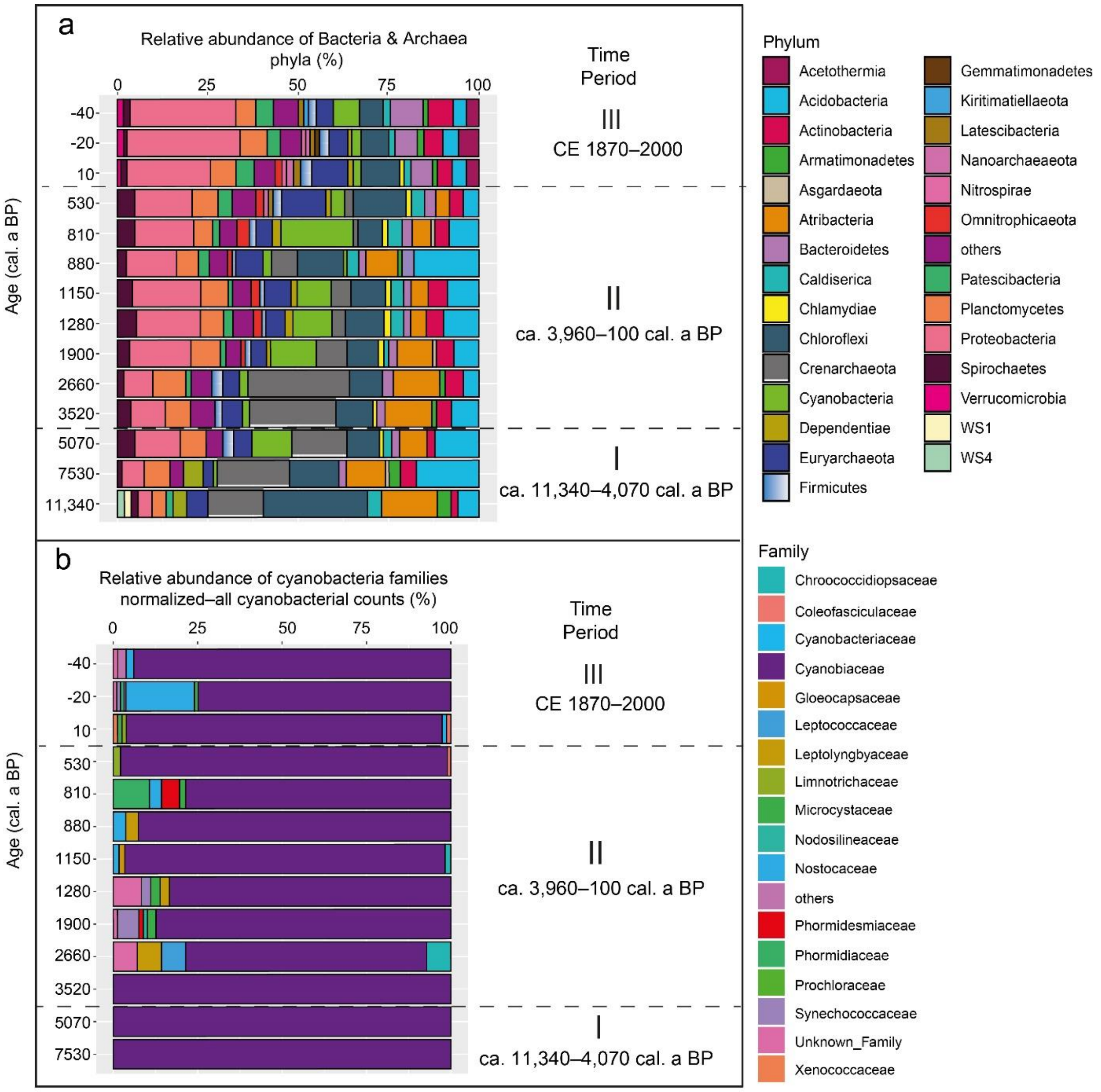

Taxonomic composition of cyanobacteria at family level based on shotgun data spanning the last ca. 11,400 years. The cyanobacterial relative abundance was normalized by the total read counts of the Bacteria domain in each sample. All families with a relative abundance below 1% were grouped into “others”. Dashed black lines demarcate the three significantly different temporal clusters identified by non-metric multidimensional scaling (see Figure 4 in main manuscript).

Supplementary Figure 5

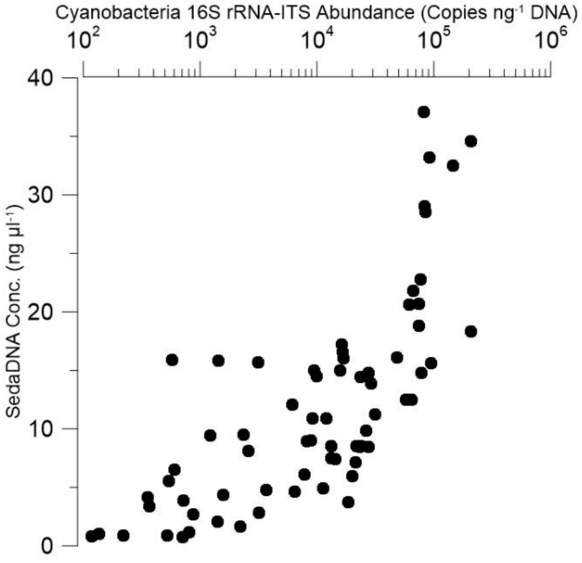

Plot to visualise the relationship between DNA extracted from sediments and total cyanobacteria abundance (16S rRNA-ITS) copy numbers quantified via qPCR assay.

Supplementary Figure 6

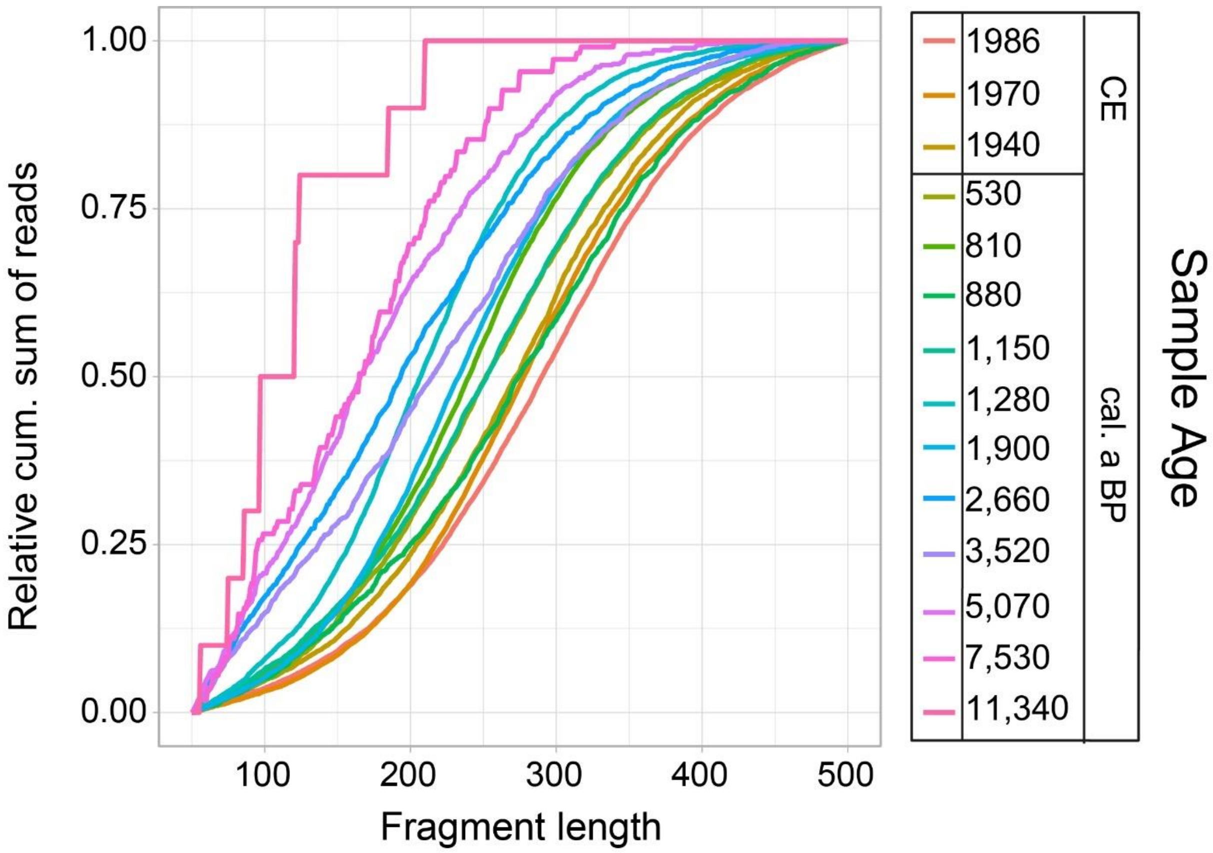

Cyanobacteria ancient DNA reads from shotgun data. Relative cumulative sum over the cyanobacteria fragment sequence length calculated from mapDamage2.0 shotgun sequence output.

Supplementary references

1. Epp, L. S., Zimmermann, H. H. & Stoof-Leichsenring, K. R. Sampling and extraction of ancient DNA from sediments. in *Methods in Molecular Biology* (eds. Shapiro B., Barlow A., Heintzman P., Hofreiter M., Paijmans J., Soares A.) **1963,** 31–44. (Humana Press, 2019). https://doi.org/10.1007/978-1-4939-9176-1_5

2. Liu, A., Zhu, T., Lu, X. & Song, L. Hydrocarbon profiles and phylogenetic analyses of diversified cyanobacterial species. *Appl. Energy* **11,** 383–393 (2013).

3. Coates, R. C. *et al.* Characterization of cyanobacterial hydrocarbon composition and distribution of biosynthetic pathways. *PLoS One* PLoS One. **9,** e85140 (2014). https://doi.org/10.1371/journal.pone.0085140

4. Bauersachs, T., Talbot, H. M., Sidgwick, F., Sivonen, K. & Schwark, L. Lipid biomarker signatures as tracers for harmful cyanobacterial blooms in the Baltic Sea. *PLoS One* **12,** e0186360 (2017).

5. Kaiser, J. *et al.* Reconstructing N2-fixing cyanobacterial blooms in the Baltic Sea beyond observations using 6- And 7-methylheptadecane in sediments as specific biomarkers. *Biogeosciences* (2020) https://doi.org/10.5194/bg-17-2579-2020.

6. Dräger, N. *et al.* Varve microfacies and varve preservation record of climate change and human impact for the last 6000 years at Lake Tiefer See (NE Germany). *The Holocene* **27**, 450–464 (2017).

7. Kaiser, J., Ön, B., Arz, H. & Akçer-Ön, S. Sedimentary lipid biomarkers in the magnesium rich and highly alkaline lake Salda (South-western anatolia). *J. Limnol.* **75,** (2016).
