# Supplementary material for: Early human impact on lake cyanobacteria revealed by a Holocene record of sedimentary ancient DNA
Source: Commun Biol. 2023 Jan 18;6:72. doi: 10.1038/s42003-023-04430-z (PMC9849356; doi:10.1038/s42003-023-04430-z)
Supplement: Supplementary file 3 — Reporting Summary [file 42003_2023_4430_MOESM3_ESM.pdf]

nature portfolio

Corresponding author(s): Ebuka Canisius Nwosu

Last updated by author(s): Dec 15, 2022

Reporting Summary

Nature Portfolio wishes to improve the reproducibility of the work that we publish. This form provides structure for consistency and transparency in reporting. For further information on Nature Portfolio policies, see our Editorial Policies and the Editorial Policy Checklist.

Statistics

For all statistical analyses, confirm that the following items are present in the figure legend, table legend, main text, or Methods section.

n/a	Confirmed	
☐	☒	The exact sample size (*n*) for each experimental group/condition, given as a discrete number and unit of measurement
☐	☒	A statement on whether measurements were taken from distinct samples or whether the same sample was measured repeatedly
☐	☒	The statistical test(s) used AND whether they are one- or two-sided<br>*Only common tests should be described solely by name; describe more complex techniques in the Methods section.*
☒	☐	A description of all covariates tested
☐	☒	A description of any assumptions or corrections, such as tests of normality and adjustment for multiple comparisons
☐	☒	A full description of the statistical parameters including central tendency (e.g. means) or other basic estimates (e.g. regression coefficient) AND variation (e.g. standard deviation) or associated estimates of uncertainty (e.g. confidence intervals)
☐	☒	For null hypothesis testing, the test statistic (e.g. *F*, *t*, *r*) with confidence intervals, effect sizes, degrees of freedom and *P* value noted<br>*Give P values as exact values whenever suitable.*
☒	☐	For Bayesian analysis, information on the choice of priors and Markov chain Monte Carlo settings
☒	☐	For hierarchical and complex designs, identification of the appropriate level for tests and full reporting of outcomes
☐	☒	Estimates of effect sizes (e.g. Cohen's *d*, Pearson's *r*), indicating how they were calculated

Our web collection on statistics for biologists contains articles on many of the points above.

Software and code

Policy information about availability of computer code

Data collection	No software was used to collect the data published in this study.
Data analysis	The amplicon sequence variants (ASVs) were generated using the DADA2 package v1.20 with the following parameters: truncLen=c(240,200), maxN=0, rm.phix=TRUE, compress=TRUE, multithread=TRUE, minLen = 150 with R v4.1. Taxonomic assignment was performed using DADA2 and SILVA database v138. The raw shotgun reads were processed using the ATLAS metagenome pipeline in order to obtain dereplicated, quality-controlled, and trimmed reads. These reads were mapped to the SILVA 16S SSU database v138 in order both to obtain taxonomy and to calculate species abundance. Mapping was performed using bowtie2 v2.4.2. In order to obtain nitrogen fixation (nif gene family) and microcystin synthesis (mcy gene family) gene abundances, quality-controlled reads were assembled into contigs using the ATLAS metagenome pipeline. The genes were predicted using prodigal v2.6.3 and annotated using the eggNog emapper v2.0.1 with a database from October 2020. TaxonKit v0.8.0 was used to obtain lineage information from taxon IDs of annotated genes. In order to obtain the final gene abundances, quality-controlled reads were mapped against all assembled contigs using bowtie2 v2.4.2. Alpha- and beta-diversity estimations as well as multivariate permutational analyses of variance (PerMANOVA) based on amplicon data were performed using the PAST v4.01 software. R version 3.3.2 using publicly available R packages all listed in the methods section. Statistical analysis of shotgun sequence data was performed using R v3.6.2 with the clusterProfile 3.14.3 package.

For manuscripts utilizing custom algorithms or software that are central to the research but not yet described in published literature, software must be made available to editors and reviewers. We strongly encourage code deposition in a community repository (e.g. GitHub). See the Nature Portfolio guidelines for submitting code & software for further information.

Data

Policy information about availability of data

All manuscripts must include a data availability statement. This statement should provide the following information, where applicable:

- Accession codes, unique identifiers, or web links for publicly available datasets
- A description of any restrictions on data availability
- For clinical datasets or third party data, please ensure that the statement adheres to our policy

The amplicon and shotgun sequencing raw reads have been uploaded to the European Nucleotide Archive (ENA) under BioProject accession number PRJEB51951. The filtered sedaDNA datasets analysed during this study are available in Supplementary Tables 1 and 2. The cyanobacteria lipid biomarker 7-methylheptadecane data analysed for this study are available in Supplementary Table 2. The age-depth model and pollen-based vegetation openness data of the last 6,000 years for Lake Tiefer See are available at https://doi.pangaea.de/10.1594/PANGAEA.862115.

Human research participants

Policy information about studies involving human research participants and Sex and Gender in Research.

Reporting on sex and gender	The study did not involve human research participants.
Population characteristics	The study did not involve human research participants.
Recruitment	The study did not involve human research participants.
Ethics oversight	The study did not involve human research participants.

Note that full information on the approval of the study protocol must also be provided in the manuscript.

Field-specific reporting

Please select the one below that is the best fit for your research. If you are not sure, read the appropriate sections before making your selection.

☒ Life sciences ☐ Behavioural & social sciences ☐ Ecological, evolutionary & environmental sciences

For a reference copy of the document with all sections, see nature.com/documents/nr-reporting-summary-flat.pdf

Life sciences study design

All studies must disclose on these points even when the disclosure is negative.

Sample size	We attempted to trace early signals of human impact on cyanobacteria structure and abundance throughout the Holocene by collecting two new overlapping sequences of 2-m long sediment cores obtained from borehole H (6 cores: TSK19_H1-H6) and borehole K (5 cores: TSK19_K1-K5) in the deepest part (62 m) of Lake Tiefer See, NE Germany. We chose our lake based on availability of other long-term paleolimnological proxy lake data such as pollen-based reconstructed vegetation openness, a proxy of human deforestation, and accessibility for sampling.
Data exclusions	Next generation sequencing: the 16S sequence reads which did not pass the preestablished quality check and filtering steps are discarded. In the analyses to account for variation in sequencing depth (e.g. for estimating beta-diversity), the amplicon sequence variant (ASV) cut-off was set to 0.1% in order to eliminate very rare taxa. The ASVs below the established threshold were discarded. Statistical analyses: no outlier was found and no data points have been excluded from analyses.
Replication	This study did not include any experiment. Replication was achieved in the survey by carrying out three DNA extractions for each sample which were pooled after extraction, and in PCR amplification steps, 2 separate PCR reaction on the same DNA extract were performed and pooled. The quantitative PCR reactions were performed in triplicates. The estimation of DNA concentration of samples for shotgun sequencing was performed twice and the average calculated.
Randomization	Our study was a survey, in which no randomisation was applied to sample collection or analysis.
Blinding	Blinding was not possible due to the nature of the samples. Because we work with time-series of DNA samples collected in sediments of various age, we must follow strict procedures to avoid contamination of old samples with modern DNA. Therefore, it is advised to process the old and recent samples separately to help reduce the risks of cross-contamination, especially during DNA extractions. For the PCR and qPCR step, all samples are treated the same way, i.e., the plates are prepared with both old and recent samples together.

Reporting for specific materials, systems and methods

We require information from authors about some types of materials, experimental systems and methods used in many studies. Here, indicate whether each material, system or method listed is relevant to your study. If you are not sure if a list item applies to your research, read the appropriate section before selecting a response.

Materials & experimental systems

n/a	Involved in the study
☒	☐ Antibodies
☒	☐ Eukaryotic cell lines
☒	☐ Palaeontology and archaeology
☒	☐ Animals and other organisms
☒	☐ Clinical data
☒	☐ Dual use research of concern

Methods

n/a	Involved in the study
☒	☐ ChIP-seq
☒	☐ Flow cytometry
☒	☐ MRI-based neuroimaging
